# Genetic proxies for therapy of insulin drug targets and risk of osteoarthritis: a drug-target Mendelian randomization analysis

**DOI:** 10.1007/s10787-024-01542-8

**Published:** 2024-08-11

**Authors:** Ziqin Cao, Qiangxiang Li, Jianhuang Wu, Yajia Li

**Affiliations:** 1 Department of Spine Surgery and Orthopaedics, Xiangya Hospital, Central South University, Changsha, China; 2 National Clinical Research Center for Geriatric Disorders, Xiangya Hospital, Central South University, Xiangya Road 87, Changsha, China; 3 Department of Dermatology, Xiangya Hospital, Central South University, Changsha, 410011 Hunan China

**Keywords:** Insulin, Osteoarthritis, Blood glucose, Drug target, Mendelian randomization

## Abstract

**Background:**

The potential effects of insulin therapy on osteoarthritis (OA) risk are poorly understood. This study aimed to explore the causal relationship between insulin therapy and OA.

**Methods:**

Mendelian randomization (MR) analysis was performed to examine the association between genetically proxied inhibition of insulin targets and the risk of overall, hip (HOA) and knee OA (KOA). We then performed univariable MR using summary statistics regarding insulin target genes derived from the DrugBank database. Data related to blood glucose reduction levels were used as a proxy for insulin levels. Two phenotypes, type 2 diabetes, and glycosylated hemoglobin levels, were selected as positive controls to confirm the direction and validity of the proxies. The OA datasets were derived from the UK Biobank cohort. Multivariable MR was adjusted for body mass index, sedentary behavior, cigarette smoking, frequency of alcohol intake, age, and genetic sex.

**Results:**

Genetically proxied insulin therapy was associated with an increased risk of overall OA [odds ratio (OR):1.2595; 95% confidence interval (CI):1.0810–1.4675] and HOA (OR:1.4218; 95%CI:1.1240–1.7985), which remained consistent across multiple MR methods. After adjusting for confounders, we found evidence supporting a significant causal link with a higher risk of overall OA and HOA. A further two-step MR analysis revealed no significant mediation effects from the six mediators in the associations.

**Conclusion:**

There was a causal association between genetically proxied insulin therapy and a higher risk of OA, especially HOA.

**Supplementary Information:**

The online version contains supplementary material available at 10.1007/s10787-024-01542-8.

## Introduction

Osteoarthritis (OA) is a chronic inflammatory joint disease that affects over 300 million individuals worldwide, representing a significant public health burden (GBD Diseases and Injuries Collaborators 2018). Mainstream therapy primarily consists of pain management and, in severe cases, joint replacement (Chen et al. 2012). First-line therapeutic drugs include nonsteroidal anti-inflammatory drugs (NSAIDs) and acetaminophen. However, OA often leads to chronic disability due to pain and impaired joint function, and many patients receiving drug therapy fail to experience significant improvements in physical function and quality of life (Bennell et al. 2012; Towheed et al. 2006).

The pathogenesis of OA is multifactorial, involving mechanical, genetic, and metabolic factors. Among these, the relationships between obesity-related insulin resistance, diabetes mellitus, and OA are critical areas of current research. Endogenous insulin secretion responds to metabolic signals that prompt pancreatic β cells to secrete the precise amount of insulin needed to maintain euglycemia. Insulin therapy for diabetes aims to replicate this complex physiological process (Kramer et al. 2021). Current pharmacotherapies, such as NSAIDs and antioxidants, have shown effectiveness in mitigating the inflammatory effects induced by insulin resistance. These drugs work by inhibiting the activity of Tumor Necrosis Factor (TNF)-α and Interleukin (IL)-6, which are known to play critical roles in the pathogenesis of osteoarthritis, thereby reducing inflammation and preventing further cartilage degradation. For instance, NSAIDs are widely used to manage pain and inflammation in OA by blocking the cyclooxygenase (COX) enzymes, which play a role in the synthesis of pro-inflammatory prostaglandins (Hotamisligil 2006). Moreover, antioxidants such as curcumin have also been shown to possess anti-inflammatory properties that can be beneficial in OA management.

However, insulin therapy has been identified to be associated with potential adverse effects, including articular cartilage degeneration in 82% of OA patients with elevated insulin levels (Ribeiro et al. 2016; Veronese et al. 2019). Insulin has been shown to increase the production of pro-inflammatory cytokines such as TNF-α and IL-6. The chronic low-grade inflammation associated with insulin resistance exacerbates the degradation of joint cartilage (Boden 2011; Kapoor et al. 2011; Scheller et al. 2014). Insulin resistance is linked to increased oxidative stress and the activation of matrix metalloproteinases (MMPs), which contribute to the breakdown of cartilage in osteoarthritis. This process highlights the intersection between metabolic dysfunction and joint degeneration (Ribeiro et al. 2016). Despite this, clinical and in vitro findings on the relationship between insulin and OA risk are inconsistent, with some studies reporting null or protective associations (Cai et al. 2002; Courties and Sellam 2016). Interpreting epidemiological data is challenging due to several factors. Pharmacoepidemiological studies are often affected by residual confounders caused by unmeasured or imprecisely measured variables, including those related to indication (McMahon 2003). Additionally, prior medication use can influence study covariates, resulting in bias due to “survivors” in the early period of pharmacotherapy (Ray 2003). Detection bias, caused by stricter monitoring and clinical tests for patients in the therapeutic group, can also produce upward bias in this group.

Further research leveraging Mendelian randomization (MR) can enhance our understanding of how genetic variants influence the effectiveness of pharmacotherapies. Personalized treatment strategies can be developed by identifying individuals who are genetically predisposed to higher inflammatory responses due to insulin resistance. Therefore, this MR analysis utilizes natural variations in genes encoding drug targets to serve as proxies for targets of insulin therapy, thereby examining the impact of therapy manipulation on disease outcomes (Smith and Ebrahim 2003). These proxies are less likely to be affected by confounders and reverse causation. MR analysis allows for the examination of long-term modulation of drug targets on OA risk, including overall OA, knee OA (KOA), and hip OA (HOA). Drug-target MR mimics the pharmacological modulation of a drug target in clinical trials, making it a useful tool for predicting the potential benefits and adverse effects of clinical therapy interventions (Ference et al. 2015; Plenge et al. 2013; Swerdlow et al. 2015). In this study, we applied an MR approach to investigate the effect of long-term inhibition of insulin therapy drug targets on the risk of overall OA, KOA, and HOA.

## Methods

### Study design

**Fig. 1:**
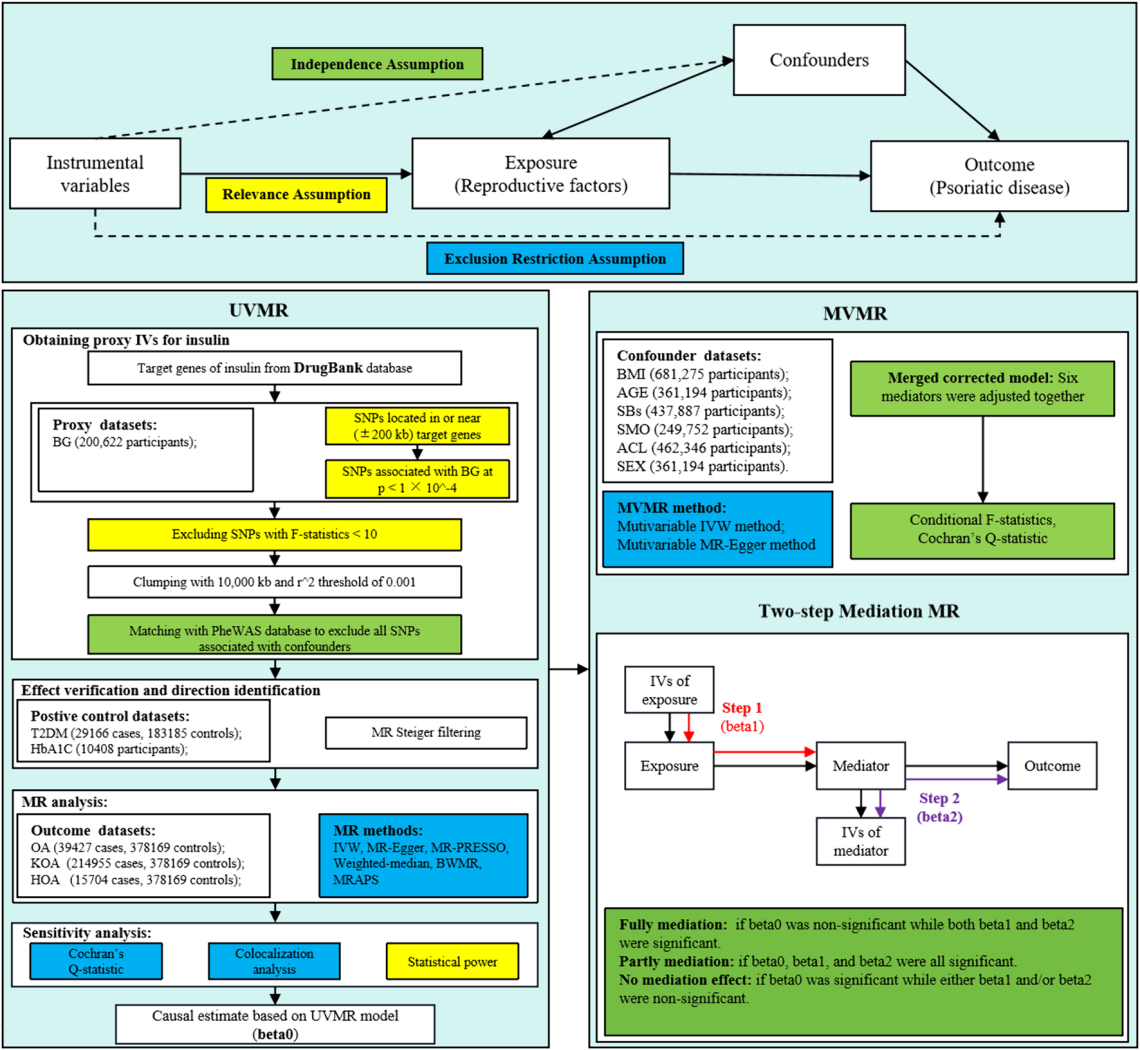
Analysis Process and Verification of Key MR Assumptions Flowchart. We utilize different colors to represent the relationship between the analysis methods and the three core assumptions of MR. Yellow indicates the relevance assumption and the methods used to verify it; green represents the independence assumption and the methods used to verify it; blue denotes the exclusion restriction assumption and the methods used for its verification. *UVMR* univariable Mendelian randomization, *MVMR* multi-variable Mendelian randomization, *BG* blood glucose levels, *T2DM* type 2 diabetes, *HbA1C* glycated hemoglobin levels, *OA* osteoarthritis, *KOA* knee osteoarthritis, *HOA* hip osteoarthritis, *ACL* frequency of alcohol intake, *AGE* age at recruitment, *BMI* body mass index, SBs sedentary behaviors, *SEX* genetic sex, *SMO* cigarette smoking

This research was conducted in accordance with the Strengthening the Reporting of Observational Studies in Epidemiology-Mendelian Randomization (STROBE-MR) guidelines. The causal relationships between genetic proxies for insulin targets and OA subtypes were assessed using a drug-target MR approach. Three fundamental assumptions were followed: (1) Relevance: The instrumental variables (IVs) were strongly correlated with exposure; (2) Exclusion Restriction: The relationship between the IVs and the outcome is exclusively mediated by exposure; (3) Independence: IVs are independent of unobserved confounding factors. Genetic variants of target genes linked to blood glucose (BG) reduction were identified and used as proxies for drug-target effects. Two phenotypes, type 2 diabetes and glycosylated hemoglobin levels, were selected as positive controls to confirm the direction and validity of the proxies. The causal impact of genetic proxies on OA and its subtypes was evaluated using various MR techniques. All data utilized in this study were publicly accessible, summarized, and validated by the IEU openGWAS (https://gwas.mrcieu.ac.uk/) and GWAS catalog databases (https://www.ebi.ac.uk/) and did not require additional ethical approval. The detailed research flowchart is presented in Fig. 1.

### Data sources and instrumental variable selection

The largest datasets of OA and its subtypes from the openGWAS database, derived from the UK Biobank cohort (Tachmazidou et al. 2019; Yengo et al. 2018), were used as outcomes to enhance statistical power. OA diagnosis was based on clinical evidence of disease requiring joint replacement or radiographic evidence of the disease (Kellgren-Lawrence grade ≥ 2). A total of 417,596 participants, comprising 39,427 patients and 378,169 controls, were included in the OA analysis. The KOA cohort included 403,124 participants, with 24,955 cases and 378,169 controls, and the HOA cohort included 393,873 participants, with 15,704 cases and 378,169 controls, respectively.

T2DM data included 212,351 participants, 29,166 cases, and 183,185 controls from the FinnGen Biobank Analysis Consortium database (https://finngen.gitbook.io/documentation/). Diagnosis was based on the International Classification of Diseases (ICD-10) criteria. The HbA1c dataset was obtained from a European cohort in a transancestral GWAS study (J. Chen et al. 2021), comprising 146,806 participants.

Considering the complex clinical and genetic background and pathogenesis of OA, we further used multivariable MR (MVMR) and two-step mediation MR analyses to validate the Independence Assumption. Since we used BG as a proxy to obtain IVs, six potential confounders known to be associated with both OA and blood glucose levels (Cao et al. 2023; Leyland et al. 2021; Szilagyi et al. 2022) were incorporated into a MVMR and mediation MR model to adjust for mediation effects. These included body mass index (BMI; sample of 681,275 participants) (Yengo et al. 2018), sedentary behaviors (SBs, 437,887 participants) (van de Vegte et al. 2020), cigarette smoking (SMO, 249,752 participants) (Liu et al. 2019), frequency of alcohol intake (ACL, 462,346 participants), age at recruitment (AGE, 361,194 participants), and genetic sex (SEX, 361,194 participants). ACL, AGE, and SEX data were sourced from the UK Biobank. Data for all mediators were collated from different databases with those used for outcome to avoid sample overlap impacts.

Information regarding target genes with defined pharmacological actions was acquired from the DrugBank database (Wishart et al. 2018). Data related to BG levels were used as proxies for insulin effects, with a sample size of 200,622. SNPs located in or near (± 200 kb) the corresponding target genes, which were associated with BG at a significance level of p < 1 × 10^−4^, were selected as proxies for insulin as described previously (Huang et al. 2021). F-statistics were calculated for each IVs and only those with F > 10 were included to minimize weak instrumental bias (Shim et al. 2015). SNPs were clumped according to a liberal linkage disequilibrium (LD) threshold of r^2^ < 0.1, using the European reference panel from the 1000 Genomes Project. MR Steiger filtering was used to exclude any IVs accounting for lower variance in the outcome than in the exposure and to determine a causal direction for a given SNP between exposure and outcome by comparing the proportion of variance explained in each (Hemani et al. 2017). Data were cross-matched with the phenome-wide association studies database (pheWAS) using a threshold of p < 5 × 10^-6 to mitigate potential links between SNPs and confounding factors. All estimates are presented per one standard deviation (SD) unit increase, and effect sizes are presented as odds ratios (OR) with a 95% confidence interval (CI). All study participants were of European ancestry to control for potential mixed population effects.

### Statistical approach

R software (version 4.1.3) with R packages, Two-Sample MR (version 0.5.6)(Hemani et al. 2018), MRPRESSO (version 1.0)(Verbanck et al. 2018), MVMR (version 0.3.0)(Sanderson et al. 2021), and MendelianRandomization (version 0.5.1)(Sanderson et al. 2021; Yavorska and Burgess 2017) were used. When an outcome association SNP was missing in the harmonization process of the IVs, a proxy with r^2^ ≥ 0.8 was used, and SNPs without proxies were removed. Six statistical methods were employed to assess the causal effects of insulin and OA in univariable MR (UVMR). The primary method was inverse-variance weighted (IVW)(Slob and Burgess 2020), which combines Wald estimates of causality for each IV to provide a relatively accurate assessment with a precise confidence interval, even under the assumption of invalid genetic instruments (Burgess et al. 2013; Staley and Burgess 2017). The MR-Egger method quantifies pleiotropy across IVs using the slope and intercept of the MR-Egger regression and offers an adjusted, robust estimate independent of IV validity (Bowden et al. 2015; Burgess and Thompson 2017). The MR-PRESSO method identifies and adjusts for distorted outliers that contribute to significant pleiotropy and heterogeneity, providing a corrected causal-effect estimate (Verbanck et al. 2018). The weighted-median method yields consistent, valid inferences when over 50% of the instrumental variables are valid (Bowden et al. 2016). Bayesian Weighted Mendelian Randomization (BWMR) corrects for pleiotropy violations and polygenic weak effect uncertainties within a Bayesian weighting framework to give reliable causal inferences (Zhao et al. 2020). The MR-Robust Adjusted Profile Score (MRAPS) method increases the statistical power to provide robust estimates under conditions of significant weak instrumental bias and horizontal pleiotropy (Zhao et al. 2018). Bonferroni correction was applied with a corrected p < 0.0083 considered significant in UVMR to prevent multiple comparison errors. Four methods, IVW, MR-Egger, MR-PRESSO, and MRAPS, were used to analyze the positive control group, and an independent Bonferroni corrected significance threshold of p < 0.0125 was adopted.

Multivariable random-effects IVW and MR-Egger methods were employed within the MVMR model, and all six mediators were concurrently adjusted to derive a consolidated, corrected evaluation. Two-step mediation MR analysis, based on different methods, quantifies the mediation effects and mechanisms for each mediator (Sanderson 2021). The total effect (beta0) denotes the direct causal impact of exposure on the outcome and is derived using the IVW method employed in UVMR. The step1 effect (beta1) indicates the causal effect value of the exposure on the mediator, and the step2 effect (beta2) represents the causal effect value of the mediator on the outcome. The association between exposure and outcome was deemed to be fully mediated by a given mediator if beta0 was non-significant, whereas both beta1 and beta2 were significant. Conversely, significant values for beta0, beta1, and beta2 indicate that the exposure-to-outcome association was partially mediated by a factor. Mediators were deemed not to influence the exposure-to-outcome association if beta0 was significant, whereas either beta1 or beta2 were non-significant. Substantial mediation effects were analyzed to identify the direct or indirect nature of the effect and calculate the proportion mediated (Burgess et al. 2017). A p-value less than 0.05 was considered statistically significant in the MVMR and mediation MR analysis.

### Sensitivity analysis

Conditional F-statistics were utilized to evaluate instrument strength in the MVMR analysis, testing the robustness of each SNP's prediction of exposure conditional on other exposures. A conditional F-value of less than 10 signifies a substantial risk of weak instrument bias.

Heterogeneity arising from the invalidity of the IVs was measured using Cochran’s Q-statistic. A p-value of less than 0.05 was deemed indicative of significant heterogeneity, and a random-effect IVW model was used if heterogeneity could not be rectified using the MR-PRESSO method (Greco et al. 2015). The MR-Egger and MR-PRESSO methods were used to test the violation of the second IV assumption, prompted by direction pleiotropy, and Cochran's Q-statistics to assess heterogeneity. Statistical power was evaluated using the binary-outcome model from the mRnd tools (https://shiny.cnsgenomics.com/mRnd/). A power below 80% indicated insufficient statistical power, and MRAPS results were prioritized. Leave-one-out analysis was conducted to identify unstable SNPs with an individual disproportionate influence on the results under the Bonferroni-corrected threshold. Such SNPs were omitted, and the results were reassessed accordingly (Burgess and Thompson 2017). Colocalization analysis using the 'coloc' R package was performed to evaluate potential violations of the exclusion restriction assumption. The likelihood of traits sharing causal variants was assessed to infer potential causality between traits that did not account for horizontal pleiotropy or the direction of the association. Thus, the probability of shared causal variants across SNP-drug target analyses was evaluated. The 'coloc' package utilizes an approximation of Bayes factor computations to produce posterior probabilities for different configurations of trait associations within a specified region. Colocalization analysis was performed within a ± 300 kb window of the top instrumental SNP for each identified drug target. Because the number of proxy SNPs was limited, reducing the statistical power, a more lenient threshold of a posterior probability greater than 0.6 was used to increase sensitivity and indicate substantial support for a specific configuration.

## Results

### Genetic instruments and validation

Six pharmacological target genes—LRP2, IGFBP7, CPE, NOV, IGF1R, and INSR—were identified in the DrugBank database. Proxy IVs were found for three loci: 134 for LRP2, 1 for IGFBP7, and 66 for IGF1R. However, no suitable SNPs within or in the vicinity of CPE, NOV, or INSR were found (S.Table 1). The positive control group was used as the reference set to align and screen proxies. Sixteen IVs with F-statistics greater than 10 were selected, suggesting a minimal risk of weak instrument bias. MR Steiger filtering indicated that all IVs maintained the correct causal direction from exposure to outcome (S.Table 2–3).

**Fig. 2:**
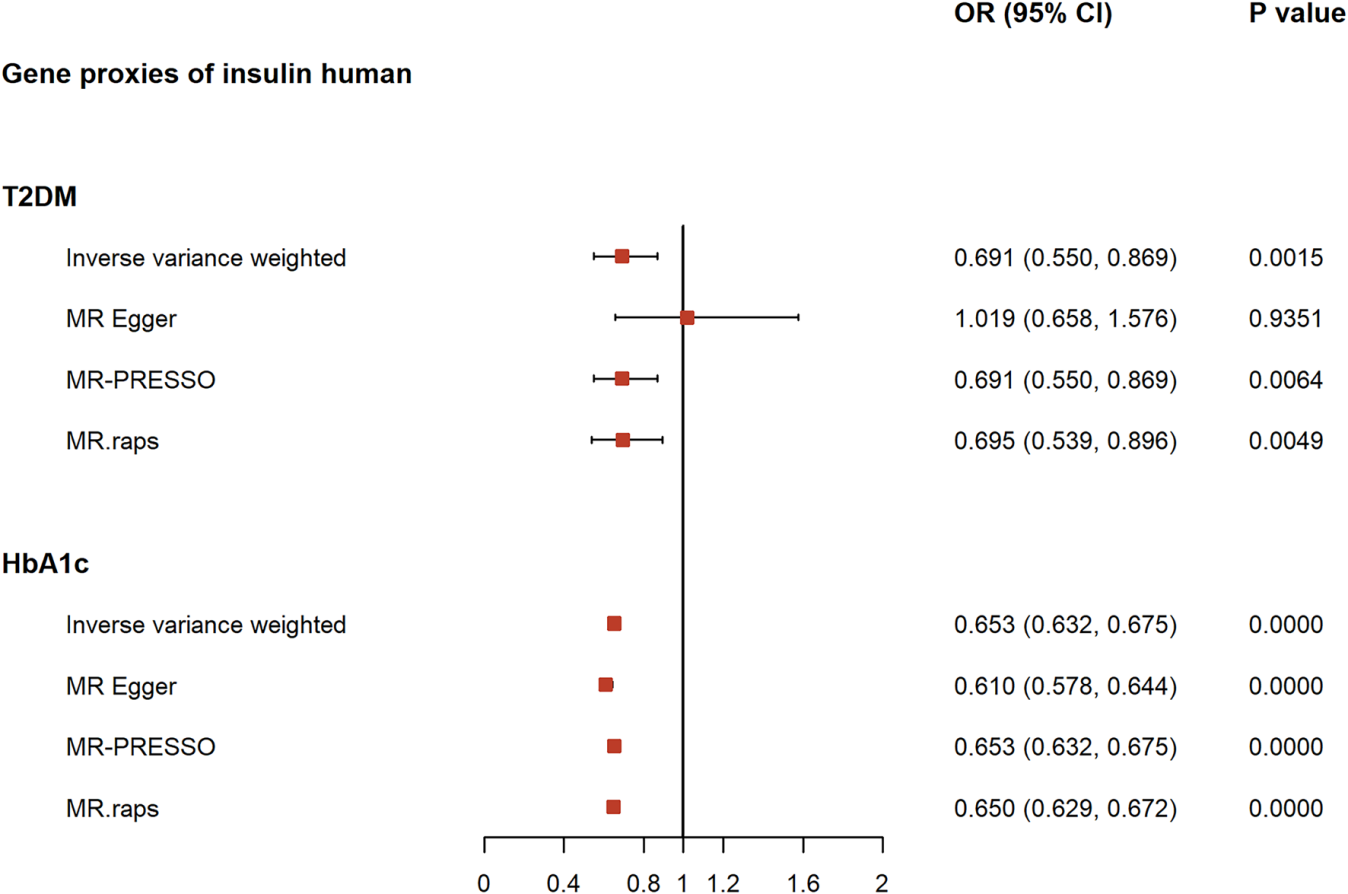
Forest plot of the univariable Mendelian randomization analyses using positive controls to confirm the direction and validity of the genetic proxies for the target gene of insulin. *OR* odds ratio, *CIs* confidence intervals, *T2DM* type 2 diabetes

Only LRP2 and IGF1R had sufficient proxy SNPs to facilitate additional colocalization analyses. The probability of shared IVs between the exposure and outcome was low (PP.H4: 0.098–0.258). Conversely, IVs were likely exclusively linked to exposure (PP.H1: 0.738–0.799). The integrity of the exclusion restriction assumption was maintained, verifying its non-violation. Details of the sensitivity analysis are presented in Table 1. No significant heterogeneity or pleiotropy was found between the insulin genetic proxies and positive controls or OA. All outcomes showed strong statistical power, ranging from 98 to 100%. Only one IV, rs508506, had a disproportionate influence over the results in the leave-one-out test of insulin and overall OA (S.Fig. 1). Scatter plots of UVMR analysis of the effects of insulin and OA are shown in S.Fig. 2.

**Table 1:** Detailed results of the sensitivity analyses. *T2DM* type 2 diabetes, *HbA1C* glycated hemoglobin levels, *OA* osteoarthritis, *KOA* knee osteoarthritis, *HOA* hip osteoarthritis.

Exposure	Outcome	Number of IVs	Heterogeneity test		MR-Egger pleiotropy test		MR-PRESSO global test				Statistics power		
			Q (P-value)	adjusted Q (P-value)	Intercept (P-value)	adjusted Intercept (P-value)	RSSobs (P-value)	adjusted RSSobs (P-value)	Heterogeneous SNPs	pleiotropic SNPs	Power	NCP	Minimum number of participants at 80% power
Insulin human	T2DM	16	15.2048 (0.4368)	NA	-0.0196 (0.0607)	NA	16.9598 (0.4770)	NA	NA	NA	100%	43.83	NA
	HbA1c	16	21.4206 (0.1239)	NA	0.0034 (0.0131)	NA	23.8109 (0.4350)	NA	NA	NA	100%	79.93	NA
Insulin human	OA	16	12.4039 (0.6482)	NA	0.0056 (0.4058)	NA	14.6390 (0.6550)	NA	NA	NA	100%	67.53	NA
	KOA	16	10.7363 (0.7711)	NA	0.0013 (0.8742)	NA	12.6628 (0.7520)	NA	NA	NA	98%	16.71	NA
	HOA	16	7.3548 (0.9470)	NA	0.0059 (0.5716)	NA	8.4606 (0.9540)	NA	NA	NA	100%	59.45	NA

The SNPs used for validation analyses of the positive controls are listed in S.Table 4. Positive control analyses showed that genetic proxies of insulin had a pronounced protective effect on both T2DM (OR_IVW_: 0.6911; 95% CI: 0.5499, 0.8687; p = 1.53E−03) and HbA1c (OR_IVW_: 0.6531; 95% CI: 0.6318, 0.6751; p = 6.32E−140). These outcomes are consistent with findings from clinical trials, validating the instrumental SNPs, as depicted in Fig. 2 and S. Table 5.

### Drug-target UVMR analysis

**Fig. 3:**
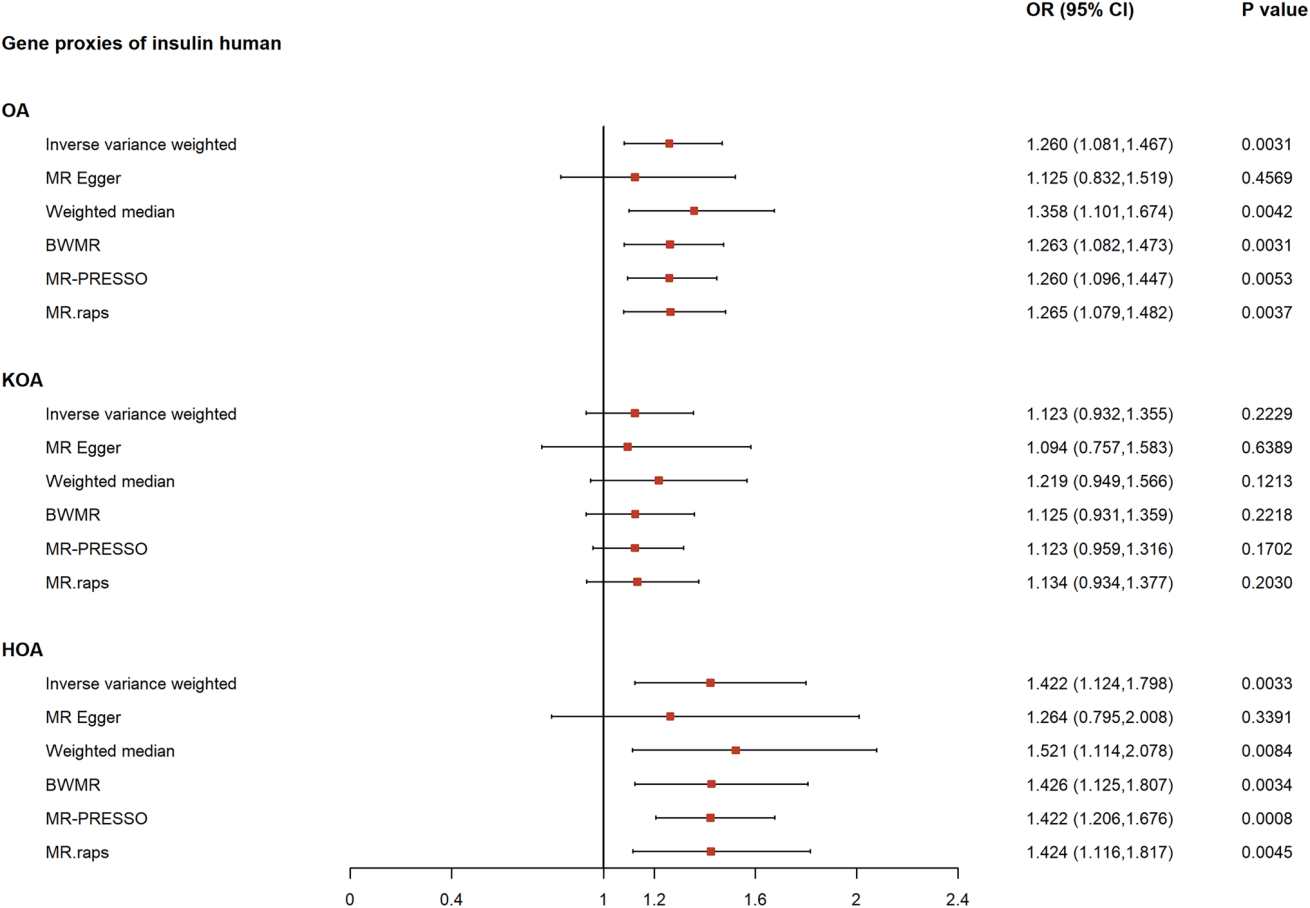
Forest plot of the univariable Mendelian randomization analyses exploring genetically determined association of the effect of insulin with osteoarthiritis and its subtypes. *OR* odds ratio, *CIs* confidence intervals, *OA* osteoarthritis, *KOA* knee osteoarthritis, *HOA* hip osteoarthritis

The accuracy of the MR-Egger method is compromised by the restricted number of SNPs, resulting in broader confidence intervals. Hence, IVW results were primarily used for UVMR, with reference to the MR-Egger findings only when considerable heterogeneity was detected. The genetic proxies for insulin showed a significant causal association with overall OA risk (OR_IVW_: 1.2595; 95% CI: 1.0810–1.4675; p = 3.08E−03), consistent with estimates from the weighted median (OR: 1.3576; 95% CI: 1.1014–1.6735; p = 4.17E−03), BWMR (OR: 1.2626; 95% CI: 1.0818–1.4735; p = 3.09E−03), MR-PRESSO (OR: 1.2595; 95% CI: 1.0961–1.4473; p = 5.33E−03), and MR.RAPS (OR: 1.2647; 95% CI: 1.0793–1.4818; p = 3.68E−03). A causal link was also detected between the genetic proxies of insulin and HOA (OR_IVW_: 1.4218; 95% CI: 1.1240–1.7985; p = 3.34E−03), which remained consistent across many MR methods. Estimates were 1.5214 (95% CI: 1.1137, 2.0785; p = 8.28E−03) by weighted median, 1.4256 (95% CI: 1.1245, 1.8072; p = 3.39E−03) by BWMR, 1.4218 (95% CI:1.2060- 1.6761; p = 7.88E−04) by MR-PRESSO, and 1.4238 (95% CI: 1.1156, 1.8172; p = 4.52E−03) by MR.RAPS. However, no significant association was found between genetic proxies for insulin and KOA (OR_IVW_: 1.1234; 95% CI: 0.9317, 1.3547; p = 2.29E−01). A forest plot of the UVMR is presented in Fig. 3**.**

### Drug-target MVMR and Mediation MR analysis

The six potential confounders were adjusted in the multivariable IVW and MR-Egger models. Weak instrumental strength was reported for the genetic proxies for insulin, with conditional F-statistics = 2.7430, likely due to the disproportionate number of IVs relative to other mediators.

**Fig. 4:**
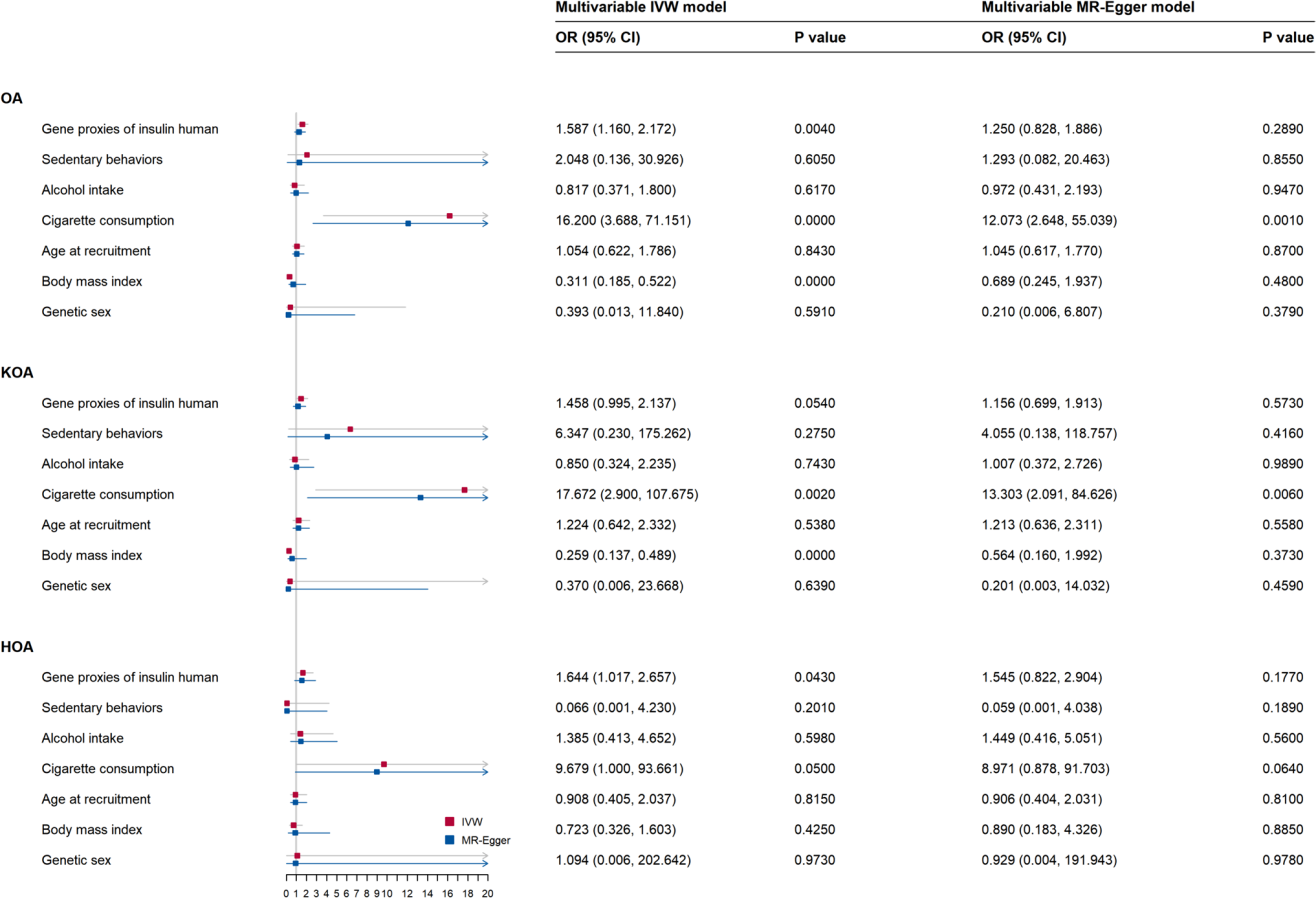
Forest plot of the multivariable Mendelian randomization analyses exploring genetically determined association of the effect of insulin with osteoarthiritis and its subtypes adjusted for confounding traits (alcohol intake, cigarettes consumption, age at recruitment, genetic sex, sedentary behaviors, and body mass index). *OR* odds ratio, *CIs* confidence intervals, *OA* osteoarthritis, *KOA* knee osteoarthritis, *HOA* hip osteoarthritis

Genetic proxies for insulin maintained a significant causal link with a higher risk of overall OA (OR_MVMR-IVW_: 1.5872; 95% CI: 1.1600, 2.1719; p = 0.004) and HOA (OR_MVMR-IVW_: 1.6438; 95% CI: 1.0169, 2.6570; p = 0.043) but not with KOA (OR_MVMR-IVW_: 1.4579; 95% CI: 0.9948, 2.1366; p = 0.0540), aligning with the UVMR findings (Fig. 4 and S.Table 6**)**. No significant mediation effects from the six mediators of the associations between genetic proxies for insulin and overall OA or HOA were detected during two-step MR analyses (Table 2), implying that the causal relationship was predominantly due to direct effects.

**Table 2:** Detailed results of the mediation MR analyses

Mediators		Outcomes: OR (95%CI), P		
		**OA**	**KOA**	**HOA**
SBs	Beta0	1.2835 (1.1017, 1.4955), 0.0014	1.1471 (0.9513, 1.3832), 0.1508	1.4532 (1.1488, 1.8383), 0.0018
	Beta1	0.9886 (0.9505, 1.0282), 0.5668	0.9886 (0.9505, 1.0282), 0.5668	0.9886 (0.9505, 1.0282), 0.5668
	Beta2	0.7392 (0.1105, 4.9446), 0.7553	0.6366 (0.0716, 5.6615), 0.6855	0.3799 (0.0411, 3.5128), 0.3938
	Direct effect	1.2791 (1.1012, 1.4857), 0.0013	1.1411 (0.9609, 1.3553), 0.1324	1.4372 (1.2059, 1.7128), 0.0001
	Mediated effect	1.0170 (0.8211, 1.2597), 0.8770	1.0235 (0.7938, 1.3197), 0.8578	1.0358 (0.7725, 1.3888), 0.8142
	Propotion of mediation	NA	NA	NA
ACL	Beta0	1.2835 (1.1017, 1.4955), 0.0014	1.1471 (0.9513, 1.3832), 0.1508	1.4532 (1.1488, 1.8383), 0.0018
	Beta1	0.9345 (0.8748, 0.9983), 0.0443	0.9345 (0.8748, 0.9983), 0.0443	0.9345 (0.8748, 0.9983), 0.0443
	Beta2	1.7099 (0.5128, 5.7013), 0.3826	1.6784 (0.4148, 6.7913), 0.4678	2.0880 (0.5005, 8.7110), 0.3124
	Direct effect	1.3310 (1.1134, 1.5912), 0.0017	1.1880 (0.9653, 1.4621), 0.1038	1.5275 (1.2354, 1.8888), 0.0001
	Mediated effect	0.9669 (0.7644, 1.2230), 0.7787	0.9728 (0.7356, 1.2864), 0.8465	0.9597 (0.6992, 1.3173), 0.7992
	Propotion of mediation	NA	NA	NA
SMO	Beta0	1.2835 (1.1017, 1.4955), 0.0014	1.1471 (0.9513, 1.3832), 0.1508	1.4532 (1.1488, 1.8383), 0.0018
	Beta1	0.9431 (0.8379, 1.0614), 0.3312	0.9431 (0.8379, 1.0614), 0.3312	0.9431 (0.8379, 1.0614), 0.3312
	Beta2	1.3617 (0.7049, 2.6308), 0.3581	1.5023 (0.6987, 3.2299), 0.2974	1.4143 (0.7036, 2.8429), 0.3300
	Direct effect	1.3070 (1.1267, 1.5161), 0.0004	1.1748 (0.9885, 1.3961), 0.0674	1.4831 (1.2664, 1.7368), 0.0000
	Mediated effect	0.9925 (0.8020, 1.2281), 0.9446	0.9777 (0.7579, 1.2612), 0.8620	1.0094 (0.7604, 1.3398), 0.9486
	Propotion of mediation	NA	NA	NA
AGE	Beta0	1.2835 (1.1017, 1.4955), 0.0014	1.1471 (0.9513, 1.3832), 0.1508	1.4532 (1.1488, 1.8383), 0.0018
	Beta1	1.7758 (1.1798, 2.6730), 0.0059	1.7758 (1.1798, 2.6730), 0.0059	1.7758 (1.1798, 2.6730), 0.0059
	Beta2	1.1061 (0.9407, 1.3005), 0.2223	1.1497 (0.9583, 1.3793), 0.1332	1.0487 (0.8566, 1.2840), 0.6449
	Direct effect	1.2113 (1.0345, 1.4183), 0.0172	1.0588 (0.8869, 1.2640), 0.5275	1.4141 (1.1600, 1.7238), 0.0006
	Mediated effect	1.0594 (0.8505, 1.3196), 0.6066	1.0827 (0.8367, 1.4010), 0.5457	1.0454 (0.7688, 1.4216), 0.7769
	Propotion of mediation	NA	NA	NA
BMI	Beta0	1.2835 (1.1017, 1.4955), 0.0014	1.1471 (0.9513, 1.3832), 0.1508	1.4532 (1.1488, 1.8383), 0.0018
	Beta1	1.0052 (0.8907, 1.1345), 0.9327	1.0052 (0.8907, 1.1345), 0.9327	1.0052 (0.8907, 1.1345), 0.9327
	Beta2	0.6479 (0.1502, 2.7951), 0.5606	0.6798 (0.0869, 5.3152), 0.7130	0.8009 (0.5826, 1.1010), 0.1710
	Direct effect	1.2864 (1.0485, 1.5784), 0.0158	1.1457 (0.1465, 8.9581), 0.8969	1.4549 (1.3916, 1.5211), 0.0000
	Mediated effect	0.9124 (0.7068, 1.1778), 0.4816	1.6875 (0.2140, 13.3073), 0.6195	0.9565 (0.7530, 1.2150), 0.7157
	Propotion of mediation	NA	NA	NA
SEX	Beta0	1.2835 (1.1017, 1.4955), 0.0014	1.1471 (0.9513, 1.3832), 0.1508	1.4532 (1.1488, 1.8383), 0.0018
	Beta1	0.9911 (0.9628, 1.0202), 0.5448	0.9911 (0.9628, 1.0202), 0.5448	0.9911 (0.9628, 1.0202), 0.5448
	Beta2	0.1767 (0.0156, 1.9946), 0.1610	0.2853 (0.0155, 5.2672), 0.3992	0.0940 (0.0055, 1.6019), 0.1020
	Direct effect	1.2638 (1.0991, 1.4532), 0.0010	1.2692 (1.0730, 1.5013), 0.0054	1.4228 (1.2082, 1.6757), 0.0000
	Mediated effect	1.0269 (0.8349, 1.2630), 0.8017	1.1498 (0.9163, 1.4429), 0.2281	1.0416 (0.7823, 1.3870), 0.7801
	Proportion of mediation	NA	NA	NA

*OR* odds ratio, *CIs* confidence intervals, *OA* osteoarthritis, *KOA* knee osteoarthritis, *HOA* hip osteoarthritis,  *ACL* frequency of alcohol intake, *AGE* age at recruitment, *BMI* body mass index, *SBs* sedentary behaviors, *SEX *genetic sex, *SMO* cigarette smoking

## Discussion

In our MR analysis of up to 39,427 OA cases and 378,169 controls, genetically proxied insulin therapy was associated with an increased risk of overall OA and HOA. This association remained significant even after adjusting for age, sex, BMI, alcohol consumption, and smoking status.

Previous studies have not directly observed this association between insulin therapy and OA risk. For example, Konstari et al. (Konstari et al. 2021) did not find that elevated plasma fasting glucose levels or diagnosed diabetes without insulin therapy predicted a higher incidence of knee OA; instead, elevated fasting plasma glucose levels were related to a reduced risk of incident knee OA. Additionally, males with diabetes, especially those requiring insulin, reported more severe joint pain and higher analgesic use compared to males without diabetes or those using other diabetes medications (Szilagyi et al. 2022). No previous study has reported an association between insulin use and HOA.

The mechanism underlying the association between genetically proxied insulin and OA risk remains unclear, and the role of insulin in OA is controversial. High insulin levels are associated with insulin resistance and T2DM (Xu et al. 2023). Distinguishing between the effects of insulin per se and insulin resistance is challenging. Excess insulin in T2DM patients may damage cartilage, and various complications are thought to result from high insulin levels in obesity and metabolic syndrome. Insulin is known to prolong chondrocyte survival, stimulate chondrocyte proliferation, and suppress differentiation, which could impair cartilage formation by inhibiting chondrocyte maturation (Alarid et al. 1992; Torres et al. 2003). Moreover, hyperinsulinemia reduces circulating T4 and its conversion to T3, mimicking hypothyroidism (Crunkhorn and Patti 2008; Farasat et al. 2012; Ortiz-Caro et al. 1984; Roos et al. 2007), and reduces the signal from thyroid hormones necessary for chondrocyte maturation, resulting in a predisposition to OA (Williams 2013).

Our study found that genetic predisposition to insulin therapy increases OA risk independently of age, sex, BMI, alcohol consumption, and smoking status. This suggests that insulin may have a direct genetic association with OA, rather than a mediated link through factors such as obesity, which predisposes individuals to OA. The inflammatory mechanisms potentially mediating this relationship may involve the upregulation of pro-inflammatory cytokines and the alteration of cartilage metabolism under chronic hyperinsulinemic conditions. Chronic hyperinsulinemia can exacerbate inflammation by promoting the production of pro-inflammatory cytokines such as IL-6 and TNF-α, which have been shown to contribute to cartilage degradation and increased synovial inflammation, important components in the pathophysiology of OA (Goldring and Otero 2011). Furthermore, insulin's impact on cartilage cells could involve adverse effects on chondrocyte proliferation and extracellular matrix production, leading to compromised joint integrity (Y. Chen et al. 2018). These insights support a more nuanced understanding of insulin's role in OA, suggesting a potential causal link between insulin therapy and increased OA risk, mediated through both metabolic and inflammatory pathways. This underscores the importance of considering the inflammatory side effects of insulin therapy in OA management and suggests avenues for therapeutic intervention that may involve modulating insulin levels or action.

There are several strengths to our MR study. First, the inclusion of participants from various European ancestries minimizes potential bias from population stratification. Additionally, various sensitivity analyses were employed to identify violations of MR assumptions. The use of summarized-level MR methods allowed us to leverage large-scale genetic information from the GWAS dataset, improving the power of the test and the reliability of causal inference. No heterogeneity was detected within the IVs in the MR analysis, indicating an absence of bias due to pleiotropy. Moreover, germline IVs were used as proxies for insulin therapy targets, facilitating the evaluation of long-term inhibition, which is a more realistic analogy of the typically decades-long use of insulin therapy compared to the short-term effects assessed in conventional observational studies and randomized trials.

Our study also has several limitations. First, only the on-target effects of insulin therapy were predicted owing to the inclusion of well-documented protein targets. Off-target drug effects not exerted by these proteins were not captured by the MR models. Second, the genetic prediction of drug effects might be different from those found in clinical therapy. An exposure instrumented by genetic variants was considered to be present from birth and last throughout the lifetime. Thus, the analyses only assessed the modulating effects of drug target proteins over the long term. Moreover, considering the lifelong genetic effects, the effects of exposure to insulin during a specific lifetime period cannot be accommodated. Third, no key gene (h4 > 0.60 was found in the colocalization analysis, suggesting that insulin-induced high OA risk was not caused by a single gene, but by the combined effects of multiple genes. The limited number of proxy SNPs meant that only LRP2 and IGF1R were included in further colocalized analysis, and CPE, NOV, INSR, and IGFBP7 with ≤ 1 SNPs were excluded. This observation may also contribute to the failure to identify key genes. The size of the BG dataset that matched the proxy SNPs was a limiting factor, and further verification would be enabled by the availability of larger and higher-quality BG datasets. Finally, the genetic data were restricted to those of European ancestry and may not be generalizable to those of different heritages.

## Conclusion

Our MR analyses suggest that genetically proxied long-term insulin therapy is associated with an increased risk of OA, particularly HOA. Evaluating insulin use in randomized controlled trials with long-term follow-up data is essential to determine the long-term safety of insulin therapy. Future studies should aim to clarify the mechanistic pathways underlying the association between insulin therapy and OA. This study also exemplifies the use of MR design to provide insights into the indications or potential contraindications for approved drugs.

## Supplementary Information

Below is the link to the electronic supplementary material.

Supplementary file1 (PDF 141 KB)

Supplementary file2 (PDF 1319 KB)

## Data Availability

The datasets used and/or analyzed during the current study are available from the corresponding author upon reasonable request.
