# Supplementary material for: Genetic proxies for therapy of insulin drug targets and risk of osteoarthritis: a drug-target Mendelian randomization analysis
Source: Inflammopharmacology. 2024 Aug 11;32(6):3717–28. doi: 10.1007/s10787-024-01542-8 (PMC11550247; doi:10.1007/s10787-024-01542-8)
Supplement: Supplementary file 1 — Supplementary file1 (PDF 141 KB) [file 10787_2024_1542_MOESM1_ESM.pdf]

Supplementary Table 1. Information of identified proxies of the drug target gene of insulin human.

Gene				SNP Proxies								
Name	Pharmacological action	Chr	Position	RS ID	Chromosome	Position	EA	OA	EAF	Beta	SE	P value
LRP2	Substrate	2	169983619:170219195	rs16856247	2	169784413	C	T	1.80E-02	0.0530	0.0058	9.09E-20
	Substrate	2	169983619:170219195	rs17540154	2	169784493	A	G	2.20E-01	0.0535	0.0021	9.25E-141
	Substrate	2	169983619:170219195	rs7607790	2	169784940	G	A	8.30E-02	0.0339	0.0030	9.29E-33
	Substrate	2	169983619:170219195	rs508506	2	169784955	A	C	6.35E-01	0.0644	0.0017	1.23E-303
	Substrate	2	169983619:170219195	rs6433096	2	169784987	A	G	8.30E-02	0.0344	0.0030	1.09E-34
	Substrate	2	169983619:170219195	rs503931	2	169785449	A	C	5.29E-01	0.0433	0.0016	2.33E-143
	Substrate	2	169983619:170219195	rs149854463	2	169785641	T	C	5.00E-03	0.0741	0.0146	3.30E-07
	Substrate	2	169983619:170219195	rs34193789	2	169786287	G	A	2.03E-01	0.0517	0.0021	2.55E-125
	Substrate	2	169983619:170219195	rs56100844	2	169786707	T	G	1.40E-02	-0.1180	0.0100	9.82E-35
	Substrate	2	169983619:170219195	rs551754	2	169787686	C	T	5.29E-01	0.0432	0.0016	2.29E-142
	Substrate	2	169983619:170219195	rs527150	2	169788039	A	G	5.29E-01	0.0432	0.0016	1.07E-142
	Substrate	2	169983619:170219195	rs114628368	2	169788429	A	G	3.40E-02	0.0264	0.0048	6.54E-08
	Substrate	2	169983619:170219195	rs531772	2	169788474	T	C	5.49E-01	0.0486	0.0016	1.51E-179
	Substrate	2	169983619:170219195	rs497692	2	169789016	T	C	5.26E-01	0.0431	0.0016	1.05E-141
	Substrate	2	169983619:170219195	rs494874	2	169789306	T	C	6.35E-01	0.0645	0.0017	4.78E-305
	Substrate	2	169983619:170219195	rs112667141	2	169789492	G	A	8.40E-02	0.0350	0.0030	9.72E-35
	Substrate	2	169983619:170219195	rs13389076	2	169789512	G	A	3.40E-02	0.0609	0.0049	1.72E-36
	Substrate	2	169983619:170219195	rs34526705	2	169789575	C	T	1.40E-01	0.0541	0.0026	1.35E-94
	Substrate	2	169983619:170219195	rs16856252	2	169789720	T	C	1.03E-01	0.0391	0.0026	9.47E-52
	Substrate	2	169983619:170219195	rs555975	2	169790935	G	A	5.22E-01	0.0425	0.0016	4.17E-138
	Substrate	2	169983619:170219195	rs552976	2	169791438	A	G	6.35E-01	0.0637	0.0017	1.46E-297
	Substrate	2	169983619:170219195	rs3755157	2	169792171	C	T	1.00E-01	0.0389	0.0026	3.98E-51
	Substrate	2	169983619:170219195	rs3755158	2	169792188	C	G	1.03E-01	0.0395	0.0027	4.83E-49
	Substrate	2	169983619:170219195	rs472614	2	169792421	A	G	5.28E-01	0.0431	0.0016	1.07E-142
	Substrate	2	169983619:170219195	rs78262659	2	169794111	A	G	8.00E-03	0.0677	0.0095	5.11E-14
	Substrate	2	169983619:170219195	rs565412	2	169794283	G	A	4.72E-01	-0.0431	0.0016	4.90E-142
	Substrate	2	169983619:170219195	rs567074	2	169794431	C	T	4.64E-01	-0.0458	0.0016	3.58E-162
	Substrate	2	169983619:170219195	rs479682	2	169794687	C	T	4.72E-01	-0.0432	0.0016	5.00E-143
	Substrate	2	169983619:170219195	rs480562	2	169794771	A	T	4.72E-01	-0.0432	0.0016	5.00E-143
	Substrate	2	169983619:170219195	rs482508	2	169794983	G	A	4.72E-01	-0.0432	0.0016	5.00E-143
	Substrate	2	169983619:170219195	rs508743	2	169795287	G	C	4.50E-01	-0.0460	0.0017	6.84E-155
	Substrate	2	169983619:170219195	rs58512362	2	169795467	A	G	1.03E-01	0.0368	0.0027	1.34E-42
	Substrate	2	169983619:170219195	rs7572878	2	169795529	C	T	8.00E-03	0.0663	0.0094	6.71E-14
	Substrate	2	169983619:170219195	rs2544360	2	169796187	T	C	5.40E-01	0.0474	0.0016	5.06E-174
	Substrate	2	169983619:170219195	rs2685803	2	169796285	A	G	5.30E-01	0.0442	0.0016	4.39E-151
	Substrate	2	169983619:170219195	rs2544367	2	169796288	T	C	5.30E-01	0.0441	0.0016	4.59E-150
	Substrate	2	169983619:170219195	rs2685804	2	169796772	A	G	5.30E-01	0.0443	0.0016	2.01E-151
	Substrate	2	169983619:170219195	rs2685805	2	169797060	A	G	5.30E-01	0.0443	0.0016	4.17E-152
	Substrate	2	169983619:170219195	rs115531051	2	169797062	C	T	8.00E-03	0.0683	0.0096	4.64E-14
	Substrate	2	169983619:170219195	rs2685806	2	169797304	A	G	5.30E-01	0.0442	0.0016	2.01E-151
	Substrate	2	169983619:170219195	rs2685807	2	169797526	C	T	5.38E-01	0.0467	0.0016	1.39E-168
	Substrate	2	169983619:170219195	rs2685808	2	169797640	G	T	5.32E-01	0.0442	0.0016	9.61E-151
	Substrate	2	169983619:170219195	rs1581397	2	169797652	T	C	5.30E-01	0.0443	0.0016	2.01E-151
	Substrate	2	169983619:170219195	rs2685810	2	169797732	T	C	5.30E-01	0.0442	0.0016	4.39E-151
	Substrate	2	169983619:170219195	rs2685811	2	169797927	A	G	5.30E-01	0.0442	0.0016	4.39E-151
	Substrate	2	169983619:170219195	rs77601801	2	169798033	A	G	2.00E-02	0.0517	0.0059	1.23E-17
	Substrate	2	169983619:170219195	rs140801079	2	169798159	A	G	5.00E-03	-0.1127	0.0201	8.19E-08
	Substrate	2	169983619:170219195	rs2685812	2	169798171	A	G	5.30E-01	0.0442	0.0016	4.59E-150
	Substrate	2	169983619:170219195	rs2685813	2	169798439	T	C	5.30E-01	0.0443	0.0016	9.15E-152
	Substrate	2	169983619:170219195	rs2685814	2	169798619	T	C	5.30E-01	0.0443	0.0016	4.39E-151
	Substrate	2	169983619:170219195	rs6709087	2	169799010	A	G	2.20E-01	0.0534	0.0021	2.16E-138
	Substrate	2	169983619:170219195	rs2250677	2	169799288	C	T	5.38E-01	0.0468	0.0016	4.13E-171
	Substrate	2	169983619:170219195	rs189347091	2	169800323	A	G	8.00E-03	0.0681	0.0096	4.45E-14
	Substrate	2	169983619:170219195	rs853791	2	169800428	A	G	5.12E-01	0.0470	0.0017	2.60E-161
	Substrate	2	169983619:170219195	rs853790	2	169800441	A	T	5.16E-01	0.0408	0.0017	3.13E-134
	Substrate	2	169983619:170219195	rs6724525	2	169800797	G	A	8.00E-03	0.0658	0.0094	1.34E-13
	Substrate	2	169983619:170219195	rs853789	2	169801488	A	G	6.38E-01	0.0664	0.0017	0.00E+00
	Substrate	2	169983619:170219195	rs860510	2	169801628	A	C	5.32E-01	0.0453	0.0016	1.15E-158
	Substrate	2	169983619:170219195	rs853788	2	169801905	T	C	5.33E-01	0.0455	0.0016	2.06E-160
	Substrate	2	169983619:170219195	rs6433100	2	169802241	A	C	8.00E-03	0.0654	0.0092	1.18E-13
	Substrate	2	169983619:170219195	rs853787	2	169802252	G	T	6.38E-01	0.0664	0.0017	0.00E+00
	Substrate	2	169983619:170219195	rs853786	2	169802310	G	A	5.29E-01	0.0448	0.0016	3.12E-156
	Substrate	2	169983619:170219195	rs862662	2	169802329	C	A	5.40E-01	0.0479	0.0016	1.95E-178
	Substrate	2	169983619:170219195	rs853785	2	169802594	C	T	5.32E-01	0.0454	0.0016	1.03E-159
	Substrate	2	169983619:170219195	rs16856261	2	169803568	C	T	9.60E-02	0.0369	0.0029	1.91E-40
	Substrate	2	169983619:170219195	rs853784	2	169803674	C	T	5.16E-01	0.0451	0.0016	3.40E-155
	Substrate	2	169983619:170219195	rs35973488	2	169803939	G	T	2.07E-01	0.0523	0.0021	8.78E-127
	Substrate	2	169983619:170219195	rs3897807	2	169805048	A	G	8.00E-03	0.0646	0.0092	1.68E-13
	Substrate	2	169983619:170219195	rs74668308	2	169805425	C	T	8.00E-03	0.0661	0.0094	8.83E-14
	Substrate	2	169983619:170219195	rs77872111	2	169805443	C	T	8.00E-03	0.0679	0.0096	4.45E-14
	Substrate	2	169983619:170219195	rs853783	2	169805511	A	C	5.16E-01	0.0454	0.0016	1.28E-157
	Substrate	2	169983619:170219195	rs74917339	2	169805862	C	G	8.00E-03	0.0657	0.0094	1.18E-13
	Substrate	2	169983619:170219195	rs55700007	2	169806021	T	G	1.00E-01	0.0364	0.0027	1.62E-41
	Substrate	2	169983619:170219195	rs853782	2	169806298	C	T	5.21E-01	0.0471	0.0016	2.19E-170
	Substrate	2	169983619:170219195	rs853781	2	169806321	A	G	5.21E-01	0.0473	0.0016	3.36E-172
	Substrate	2	169983619:170219195	rs7570477	2	169806724	A	G	8.00E-03	0.0653	0.0094	1.68E-13
	Substrate	2	169983619:170219195	rs72886761	2	169806738	G	A	9.90E-02	0.0369	0.0027	1.04E-42
	Substrate	2	169983619:170219195	rs7558863	2	169807362	T	C	2.10E-01	0.0525	0.0021	5.21E-128
	Substrate	2	169983619:170219195	rs853780	2	169807482	C	G	5.12E-01	0.0447	0.0016	3.93E-153
	Substrate	2	169983619:170219195	rs1101534	2	169807713	C	T	5.13E-01	0.0446	0.0016	1.90E-152
	Substrate	2	169983619:170219195	rs1101533	2	169808522	A	T	5.14E-01	0.0447	0.0016	8.64E-153
	Substrate	2	169983619:170219195	rs853779	2	169809672	G	T	5.13E-01	0.0448	0.0016	8.64E-153
	Substrate	2	169983619:170219195	rs147267401	2	169810163	T	G	1.10E-02	0.0823	0.0122	1.83E-12
	Substrate	2	169983619:170219195	rs1101532	2	169810416	T	C	5.25E-01	0.0485	0.0016	6.41E-180
	Substrate	2	169983619:170219195	rs181497079	2	169811033	T	C	1.00E-03	-0.3671	0.0928	4.63E-05
	Substrate	2	169983619:170219195	rs853778	2	169811224	T	C	5.17E-01	0.0459	0.0016	1.03E-159
	Substrate	2	169983619:170219195	rs1101531	2	169811618	T	A	5.12E-01	0.0448	0.0016	1.78E-153
	Substrate	2	169983619:170219195	rs115941163	2	169811749	C	T	1.70E-02	0.0634	0.0078	3.51E-16
	Substrate	2	169983619:170219195	rs864929	2	169812125	G	A	5.12E-01	0.0448	0.0016	1.78E-153

	Substrate	2	169983619:170219195	rs853777	2	169812217	T	C	6.21E-01	0.0669	0.0017	0.00E+00
	Substrate	2	169983619:170219195	rs114691375	2	169813318	T	A	3.70E-02	0.0452	0.0058	7.79E-17
	Substrate	2	169983619:170219195	rs853776	2	169813353	C	T	5.11E-01	0.0448	0.0016	8.09E-154
	Substrate	2	169983619:170219195	rs853775	2	169813371	G	A	5.21E-01	0.0477	0.0017	1.73E-175
	Substrate	2	169983619:170219195	rs853774	2	169813396	G	A	3.06E-01	0.0216	0.0018	1.36E-31
	Substrate	2	169983619:170219195	rs78344358	2	169813769	G	T	8.00E-03	0.0682	0.0097	8.97E-14
	Substrate	2	169983619:170219195	rs853773	2	169814347	A	G	4.86E-01	0.0489	0.0017	2.79E-183
	Substrate	2	169983619:170219195	rs853772	2	169814655	G	T	5.01E-01	0.0437	0.0017	2.35E-144
	Substrate	2	169983619:170219195	rs72886778	2	169815106	T	A	1.40E-02	0.0693	0.0124	1.73E-08
	Substrate	2	169983619:170219195	rs73018724	2	169815248	A	G	4.50E-02	0.0528	0.0056	1.35E-21
	Substrate	2	169983619:170219195	rs76031050	2	169816670	G	A	2.90E-02	-0.0560	0.0055	1.49E-24
	Substrate	2	169983619:170219195	rs3821120	2	169816936	G	C	3.39E-01	0.0324	0.0019	7.72E-61
	Substrate	2	169983619:170219195	rs3755160	2	169817403	C	T	3.14E-01	0.0250	0.0018	1.44E-41
	Substrate	2	169983619:170219195	rs16823014	2	169817713	G	A	5.00E-02	0.0258	0.0046	1.01E-08
	Substrate	2	169983619:170219195	rs4148797	2	169818035	T	C	2.66E-01	0.0222	0.0019	2.79E-30
	Substrate	2	169983619:170219195	rs16856300	2	169818302	A	C	3.51E-01	0.0152	0.0018	8.28E-16
	Substrate	2	169983619:170219195	rs3770582	2	169819824	G	A	3.50E-01	0.0143	0.0018	3.83E-16
	Substrate	2	169983619:170219195	rs4148796	2	169820618	A	G	1.70E-01	0.0226	0.0024	2.82E-20
	Substrate	2	169983619:170219195	rs4148795	2	169820619	T	C	1.69E-01	0.0224	0.0024	6.05E-20
	Substrate	2	169983619:170219195	rs17267869	2	169823408	T	C	1.74E-01	-0.0180	0.0027	1.74E-10
	Substrate	2	169983619:170219195	rs34882395	2	169826989	G	T	1.08E-01	-0.0154	0.0035	7.36E-06
	Substrate	2	169983619:170219195	rs2216502	2	169826998	A	G	6.50E-02	0.0246	0.0037	2.53E-09
	Substrate	2	169983619:170219195	rs55669065	2	169830443	C	T	1.16E-01	-0.0158	0.0033	2.07E-06
	Substrate	2	169983619:170219195	rs76213220	2	169831239	G	C	1.19E-01	-0.0162	0.0033	1.03E-06
	Substrate	2	169983619:170219195	rs2287619	2	169836730	T	C	6.50E-02	0.0174	0.0031	2.06E-07
	Substrate	2	169983619:170219195	rs62171035	2	169838304	C	T	6.30E-02	0.0185	0.0033	5.49E-07
	Substrate	2	169983619:170219195	rs62171037	2	169843540	C	T	5.70E-02	0.0250	0.0042	1.21E-07
	Substrate	2	169983619:170219195	rs185719303	2	169848229	C	A	3.00E-03	-0.0938	0.0208	6.37E-06
	Substrate	2	169983619:170219195	rs78347243	2	169848557	C	T	6.30E-02	0.0179	0.0033	1.41E-06
	Substrate	2	169983619:170219195	rs62171038	2	169857441	C	T	6.60E-02	0.0184	0.0033	3.33E-07
	Substrate	2	169983619:170219195	rs62171039	2	169857628	T	A	6.60E-02	0.0185	0.0033	3.07E-07
	Substrate	2	169983619:170219195	rs62171040	2	169858061	G	A	5.30E-02	0.0258	0.0042	2.94E-08
	Substrate	2	169983619:170219195	rs62171051	2	169859166	A	G	6.60E-02	0.0185	0.0033	2.82E-07
	Substrate	2	169983619:170219195	rs78896123	2	169865452	C	T	1.40E-02	0.0611	0.0137	5.48E-05
	Substrate	2	169983619:170219195	rs3770601	2	169872644	C	T	6.70E-02	0.0150	0.0031	7.47E-06
	Substrate	2	169983619:170219195	rs4148773	2	169873821	G	A	6.70E-02	0.0161	0.0033	7.26E-06
	Substrate	2	169983619:170219195	rs3770603	2	169883218	C	T	6.70E-02	0.0165	0.0033	3.87E-06
	Substrate	2	169983619:170219195	rs58830528	2	169884535	T	G	6.70E-02	0.0165	0.0033	5.22E-06
	Substrate	2	169983619:170219195	rs62171052	2	169885980	G	A	6.70E-02	0.0165	0.0033	4.85E-06
	Substrate	2	169983619:170219195	rs4148768	2	169887154	G	A	6.70E-02	0.0164	0.0033	5.22E-06
	Substrate	2	169983619:170219195	rs4148765	2	169889579	C	T	6.60E-02	0.0160	0.0034	1.71E-05
	Substrate	2	169983619:170219195	rs16856392	2	169889653	C	T	6.60E-02	0.0168	0.0033	5.08E-06
	Substrate	2	169983619:170219195	rs148766104	2	169912240	G	A	1.30E-02	-0.0414	0.0087	3.77E-07
	Substrate	2	169983619:170219195	rs142804484	2	169936727	G	T	1.40E-02	-0.0346	0.0083	1.41E-05
	Substrate	2	169983619:170219195	rs199672898	2	169939988	G	A	1.00E-03	-0.1593	0.0354	1.45E-06
IGFBP7	Inhibitor/Binder	4	57896939:57976551	rs186316818	4	58090536	C	T	9.00E-03	0.0398	0.0111	8.45E-05
CPE	Modulator	4	166282346:166419472	NA	NA	NA	NA	NA	NA	NA	NA	NA
NOV	Downregulator	8	120428546:120436593	NA	NA	NA	NA	NA	NA	NA	NA	NA
IGF1R	Activator	15	99192200:99507759	rs4966014	15	99248018	C	T	6.97E-01	-0.0093	0.0020	3.06E-06
	Activator	15	99192200:99507759	rs11858316	15	99249029	C	T	6.10E-01	-0.0104	0.0019	1.44E-09
	Activator	15	99192200:99507759	rs7174918	15	99251356	C	T	3.10E-01	0.0096	0.0020	4.91E-07
	Activator	15	99192200:99507759	rs8031839	15	99253965	G	A	6.03E-01	-0.0111	0.0019	1.88E-10
	Activator	15	99192200:99507759	rs7166890	15	99254194	C	T	3.09E-01	0.0090	0.0020	1.06E-06
	Activator	15	99192200:99507759	rs8032477	15	99254554	C	T	6.10E-01	-0.0110	0.0019	2.66E-10
	Activator	15	99192200:99507759	rs12906223	15	99255960	T	G	6.08E-01	-0.0106	0.0019	1.03E-09
	Activator	15	99192200:99507759	rs3803476	15	99256570	A	G	6.20E-01	-0.0104	0.0019	3.27E-09
	Activator	15	99192200:99507759	rs11634241	15	99259016	G	A	3.40E-01	0.0097	0.0019	3.75E-08
	Activator	15	99192200:99507759	rs45579742	15	99263236	G	C	3.05E-01	0.0085	0.0020	2.89E-06
	Activator	15	99192200:99507759	rs8038015	15	99263274	T	C	3.69E-01	0.0102	0.0019	4.51E-09
	Activator	15	99192200:99507759	rs11857366	15	99263801	A	G	6.14E-01	-0.0107	0.0018	8.74E-10
	Activator	15	99192200:99507759	rs28684460	15	99264767	C	A	3.03E-01	0.0086	0.0020	2.89E-06
	Activator	15	99192200:99507759	rs4965428	15	99267568	G	C	6.15E-01	-0.0102	0.0019	4.51E-09
	Activator	15	99192200:99507759	rs932071	15	99268030	G	A	3.05E-01	0.0082	0.0020	6.75E-06
	Activator	15	99192200:99507759	rs4616271	15	99268259	C	T	3.03E-01	0.0082	0.0020	6.75E-06
	Activator	15	99192200:99507759	rs4965430	15	99268850	C	G	6.33E-01	-0.0098	0.0019	2.17E-08
	Activator	15	99192200:99507759	rs4965431	15	99269147	G	A	5.94E-01	-0.0102	0.0020	8.13E-09
	Activator	15	99192200:99507759	rs28657002	15	99269878	T	A	2.97E-01	0.0086	0.0020	1.99E-06
	Activator	15	99192200:99507759	rs55686521	15	99270441	T	C	2.98E-01	0.0086	0.0020	1.99E-06
	Activator	15	99192200:99507759	rs6598541	15	99271135	A	G	6.48E-01	-0.0114	0.0017	4.12E-12
	Activator	15	99192200:99507759	rs62024488	15	99271685	A	G	3.03E-01	0.0087	0.0020	1.76E-06
	Activator	15	99192200:99507759	rs875686	15	99272519	T	A	2.96E-01	0.0087	0.0020	1.76E-06
	Activator	15	99192200:99507759	rs907799	15	99272663	A	G	2.96E-01	0.0087	0.0020	1.76E-06
	Activator	15	99192200:99507759	rs11633717	15	99273032	T	C	3.01E-01	0.0088	0.0020	1.36E-06
	Activator	15	99192200:99507759	rs7166287	15	99273075	C	T	6.50E-01	-0.0103	0.0019	3.27E-09
	Activator	15	99192200:99507759	rs4966019	15	99274326	C	T	6.44E-01	-0.0104	0.0019	2.36E-09
	Activator	15	99192200:99507759	rs11633294	15	99275008	C	A	2.96E-01	0.0089	0.0020	1.06E-06
	Activator	15	99192200:99507759	rs67291025	15	99276257	C	G	3.00E-01	0.0088	0.0020	1.20E-06
	Activator	15	99192200:99507759	rs62024490	15	99276293	A	G	3.00E-01	0.0089	0.0020	1.06E-06
	Activator	15	99192200:99507759	rs62024491	15	99276297	G	A	2.94E-01	0.0088	0.0020	1.36E-06
	Activator	15	99192200:99507759	rs59646751	15	99276521	G	T	3.00E-01	0.0089	0.0020	1.06E-06
	Activator	15	99192200:99507759	rs1849554	15	99279846	C	T	6.56E-01	-0.0088	0.0019	1.55E-06
	Activator	15	99192200:99507759	rs907808	15	99280254	A	G	2.94E-01	0.0084	0.0020	5.31E-06
	Activator	15	99192200:99507759	rs62024526	15	99281188	C	T	2.81E-01	0.0086	0.0020	3.56E-06
	Activator	15	99192200:99507759	rs1976668	15	99281192	C	T	6.48E-01	-0.0100	0.0019	2.40E-08
	Activator	15	99192200:99507759	rs28733344	15	99281289	A	G	2.96E-01	0.0084	0.0020	4.17E-06
	Activator	15	99192200:99507759	rs1567811	15	99281833	C	G	6.81E-01	-0.0089	0.0019	1.55E-06
	Activator	15	99192200:99507759	rs3743263	15	99282558	A	G	6.82E-01	-0.0088	0.0019	1.55E-06
	Activator	15	99192200:99507759	rs3743264	15	99282587	G	A	6.81E-01	-0.0089	0.0019	1.76E-06
	Activator	15	99192200:99507759	rs1007212	15	99283918	G	A	6.81E-01	-0.0093	0.0019	6.30E-07
	Activator	15	99192200:99507759	rs2871974	15	99284074	C	T	6.44E-01	-0.0103	0.0019	3.84E-09
	Activator	15	99192200:99507759	rs12911662	15	99284466	T	G	5.08E-01	-0.0082	0.0022	5.97E-05
	Activator	15	99192200:99507759	rs8037467	15	99284652	T	G	2.96E-01	0.0087	0.0020	1.99E-06
	Activator	15	99192200:99507759	rs4966020	15	99284680	G	A	6.48E-01	-0.0098	0.0019	4.34E-08
	Activator	15	99192200:99507759	rs4966021	15	99285056	C	T	6.41E-01	-0.0100	0.0019	6.21E-09
	Activator	15	99192200:99507759	rs45490892	15	99285115	A	G	2.96E-01	0.0081	0.0020	7.60E-06

	Activator	15	99192200:99507759	rs4966022	15	99286284	A	G	6.81E-01	-0.0088	0.0019	1.55E-06
	Activator	15	99192200:99507759	rs61168554	15	99286980	A	G	3.38E-01	0.0092	0.0019	1.82E-07
	Activator	15	99192200:99507759	rs12908437	15	99287375	T	C	6.40E-01	-0.0112	0.0017	7.60E-12
	Activator	15	99192200:99507759	rs62024530	15	99287474	G	A	2.96E-01	0.0083	0.0020	8.09E-06
	Activator	15	99192200:99507759	rs57516305	15	99288559	C	T	2.98E-01	0.0083	0.0020	8.09E-06
	Activator	15	99192200:99507759	rs59350108	15	99289607	G	C	3.28E-01	0.0095	0.0019	1.03E-07
	Activator	15	99192200:99507759	rs2137683	15	99289638	C	G	6.38E-01	-0.0102	0.0019	7.13E-09
	Activator	15	99192200:99507759	rs62024533	15	99290500	A	C	3.00E-01	0.0082	0.0020	1.14E-05
	Activator	15	99192200:99507759	rs62024534	15	99290751	T	C	2.96E-01	0.0084	0.0020	7.21E-06
	Activator	15	99192200:99507759	rs62024535	15	99290793	A	G	2.94E-01	0.0084	0.0020	7.21E-06
	Activator	15	99192200:99507759	rs11632952	15	99290975	A	G	2.96E-01	0.0083	0.0020	7.21E-06
	Activator	15	99192200:99507759	rs7166999	15	99292805	A	C	6.48E-01	-0.0099	0.0019	4.34E-08
	Activator	15	99192200:99507759	rs6598542	15	99293056	G	A	6.48E-01	-0.0102	0.0019	1.32E-08
	Activator	15	99192200:99507759	rs6598543	15	99293552	A	G	6.45E-01	-0.0099	0.0019	2.79E-08
	Activator	15	99192200:99507759	rs12898337	15	99294355	T	C	6.50E-01	-0.0100	0.0019	2.07E-08
	Activator	15	99192200:99507759	rs12898502	15	99294452	T	C	6.82E-01	-0.0089	0.0020	1.55E-06
	Activator	15	99192200:99507759	rs4966024	15	99295570	G	A	6.46E-01	-0.0099	0.0019	3.23E-08
	Activator	15	99192200:99507759	rs12911273	15	99295650	C	T	6.29E-01	-0.0096	0.0019	3.25E-07
	Activator	15	99192200:99507759	rs7182976	15	99296656	C	A	5.44E-01	-0.0075	0.0020	6.45E-05
INSR	Agonist	19	7112266:7294045	NA	NA	NA	NA	NA	NA	NA	NA	NA

Supplementary Table 2. Information of identified SNPs in the valdation analysis.

SNP					Exposure (Drug effect of insulin)						Outcome (T2DM)						Outcome (HbA1C)					
RS ID	Chromoso me	Position	EA	OA	F statistics	Beta	Se	P-value	EAF	R2 of the exposure	Beta	Se	P-value	EAF	Steriger test of causal direction	R2 of the outcome	Beta	Se	P-value	EAF	Steriger test of causal direction	R2 of the outcome
rs115941163	2	169811749	C	T	231.6914893	0.0634	0.0078	3.51E-16	0.0170	1.8144%	-0.0762	0.0479	1.12E-01	0.9865	Ture	0.3452%	0.0239	0.0060	4.55E-04	0.0170	Ture	1.0396%
rs148766104	2	169912240	G	A	131.7958759	-0.0414	0.0087	3.77E-07	0.0130	1.0624%	0.0305	0.0386	4.30E-01	0.9790	Ture	0.1715%	-0.0118	0.0064	4.71E-02	0.0130	Ture	0.4812%
rs16823014	2	169817713	G	A	158.977125	0.0258	0.0046	1.01E-08	0.0500	1.2521%	0.0300	0.0352	3.95E-01	0.9747	Ture	0.1849%	0.0101	0.0038	5.46E-03	0.0500	Ture	0.6937%
rs16856252	2	169789720	T	C	435.3281744	0.0391	0.0026	9.47E-52	0.1030	3.3556%	-0.0320	0.0177	7.00E-02	0.8956	Ture	0.3923%	0.0170	0.0020	7.14E-19	0.1030	Ture	2.2179%
rs17267869	2	169823408	T	C	183.8567458	-0.0180	0.0027	1.74E-10	0.1740	1.4882%	-0.0009	0.0164	9.58E-01	0.8733	Ture	0.0119%	-0.0084	0.0022	2.00E-04	0.1740	Ture	0.9965%
rs189347091	2	169800323	A	G	201.7383292	0.0681	0.0096	4.45E-14	0.0080	1.5836%	-0.0628	0.0826	4.47E-01	0.9959	Ture	0.1650%	0.0366	0.0073	4.34E-08	0.0080	Ture	1.3084%
rs2216502	2	169826998	A	G	188.8923625	0.0246	0.0037	2.53E-09	0.0650	1.4842%	-0.0200	0.0212	3.47E-01	0.9291	Ture	0.2047%	0.0100	0.0027	6.05E-05	0.0650	Ture	0.9666%
rs34526705	2	169789575	C	T	610.1360344	0.0541	0.0026	1.35E-94	0.1400	4.6405%	-0.0316	0.0184	8.54E-02	0.9023	Ture	0.3727%	0.0255	0.0019	4.62E-40	0.1400	Ture	3.5007%
rs4148796	2	169820618	A	G	269.1917151	0.0226	0.0024	2.82E-20	0.1700	2.1019%	-0.0165	0.0126	1.89E-01	0.7436	Ture	0.2842%	0.0093	0.0019	4.53E-06	0.1700	Ture	1.2774%
rs508506	2	169784955	A	C	1153.875809	0.0644	0.0017	1.00E-200	0.6350	8.4276%	-0.0199	0.0114	8.20E-02	0.3426	Ture	0.3788%	0.0267	0.0013	1.06E-98	0.6350	Ture	5.3527%
rs56100844	2	169786707	T	G	321.7189117	-0.1180	0.0100	9.82E-35	0.0140	2.6336%	-0.0155	0.0430	7.18E-01	0.9836	Ture	0.0782%	-0.0580	0.0071	4.47E-17	0.0140	Ture	2.1316%
rs6598541	15	99271135	A	G	184.9220657	-0.0114	0.0017	4.12E-12	0.6480	1.4970%	0.0274	0.0113	1.55E-02	0.3495	Ture	0.5262%	-0.0002	0.0013	4.30E-01	0.6480	Ture	0.0402%
rs72886778	2	169815106	T	A	158.4035653	0.0693	0.0124	1.73E-08	0.0140	1.2476%	0.0086	0.1285	9.47E-01	0.0022	Ture	0.0145%	0.0400	0.0093	1.14E-06	0.0140	Ture	1.1225%
rs73018724	2	169815248	A	G	269.5391949	0.0528	0.0056	1.35E-21	0.0450	2.1046%	-0.0690	0.0417	9.85E-02	0.9820	Ture	0.3591%	0.0263	0.0043	1.61E-08	0.0450	Ture	1.5961%
rs76031050	2	169816670	G	A	278.6047056	-0.0560	0.0055	1.49E-24	0.0290	2.2726%	-0.0112	0.0213	5.98E-01	0.9302	Ture	0.1141%	-0.0205	0.0043	8.53E-07	0.0290	Ture	1.2442%
rs78896123	2	169865452	C	T	126.0896871	0.0611	0.0137	5.48E-05	0.0140	0.9957%	-0.0049	0.0453	9.15E-01	0.9853	Ture	0.0235%	0.0201	0.0105	1.52E-01	0.0140	Ture	0.4996%

Supplementary Table 3. Information of identified SNPs in exposure and outcomes.

SNP					Exposure (Drug effect of insulin)						Outcome (OA)						Outcome (KOA)						Outcome (HOA)					
RS ID	Chromosome	Position	EA	OA	F statistics	Beta	Se	P-value	EAF	R2 of the exposure	Beta	Se	P-value	EAF	Steriger test of causal direction	R2 of the outcome	Beta	Se	P-value	EAF	Steriger test of causal direction	R2 of the outcome	Beta	Se	P-value	EAF	Steriger test of causal direction	R2 of the outcome
rs115941163	2	169811749	C	T	231.6914841	0.0634	0.0078	3.51E-16	0.0170	1.8144%	0.0634	0.0078	3.51E-16	0.0170	Ture	1.8144%	0.0425	0.0411	3.01E-01	0.0144	Ture	0.1629%	0.0469	0.0512	3.59E-01	0.0144	Ture	0.1460%
rs148766104	2	169912240	G	A	131.7958774	-0.0414	0.0087	3.77E-07	0.0130	1.0624%	-0.0414	0.0087	3.77E-07	0.0130	Ture	1.0624%	-0.0489	0.0342	1.53E-01	0.0204	Ture	0.2252%	-0.0544	0.0427	2.03E-01	0.0205	Ture	0.2030%
rs16823014	2	169817713	G	A	158.977125	0.0258	0.0046	1.01E-08	0.0500	1.2521%	0.0258	0.0046	1.01E-08	0.0500	Ture	1.2521%	0.0298	0.0199	1.34E-01	0.0607	Ture	0.2359%	0.0058	0.0250	8.16E-01	0.0606	Ture	0.0370%
rs16856252	2	169789720	T	C	435.3281743	0.0391	0.0026	9.47E-52	0.1030	3.3556%	0.0391	0.0026	9.47E-52	0.1030	Ture	3.3556%	-0.0040	0.0151	7.90E-01	0.1065	Ture	0.0417%	0.0167	0.0189	3.78E-01	0.1065	Ture	0.1408%
rs17267869	2	169823408	T	C	183.8567458	-0.0180	0.0027	1.74E-10	0.1740	1.4882%	-0.0180	0.0027	1.74E-10	0.1740	Ture	1.4882%	0.0029	0.0129	8.20E-01	0.1651	Ture	0.0354%	-0.0165	0.0161	3.07E-01	0.1647	Ture	0.1633%
rs189347091	2	169800323	A	G	201.7383302	0.0681	0.0096	4.45E-14	0.0080	1.5836%	0.0681	0.0096	4.45E-14	0.0080	Ture	1.5836%	-0.0049	0.0513	9.24E-01	0.0086	Ture	0.0150%	0.0302	0.0647	6.41E-01	0.0085	Ture	0.0744%
rs2216502	2	169826998	A	G	188.892364	0.0246	0.0037	2.53E-09	0.0650	1.4842%	0.0246	0.0037	2.53E-09	0.0650	Ture	1.4842%	-0.0060	0.0180	7.40E-01	0.0747	Ture	0.0525%	-0.0134	0.0226	5.54E-01	0.0749	Ture	0.0945%
rs34526705	2	169789575	C	T	610.1360371	0.0541	0.0026	1.35E-94	0.1400	4.6405%	0.0541	0.0026	1.35E-94	0.1400	Ture	4.6405%	0.0054	0.0132	6.79E-01	0.1483	Ture	0.0644%	-0.0007	0.0165	9.66E-01	0.1485	Ture	0.0068%
rs4148796	2	169820618	A	G	269.1917087	0.0226	0.0024	2.82E-20	0.1700	2.1019%	0.0226	0.0024	2.82E-20	0.1700	Ture	2.1019%	-0.0001	0.0107	9.93E-01	0.2524	Ture	0.0015%	0.0221	0.0134	9.92E-02	0.2516	Ture	0.2628%
rs508506	2	169784955	A	C	1153.875816	0.0644	0.0017	1.00E-200	0.6350	8.4276%	0.0644	0.0017	1.00E-200	0.6350	Ture	8.4276%	0.0155	0.0097	1.10E-01	0.6480	Ture	0.2517%	0.0270	0.0122	2.66E-02	0.6479	Ture	0.3526%
rs56100844	2	169786707	T	G	321.7189089	-0.1180	0.0100	9.82E-35	0.0140	2.6336%	-0.1180	0.0100	9.82E-35	0.0140	Ture	2.6336%	-0.0233	0.0407	5.67E-01	0.0146	Ture	0.0902%	-0.0636	0.0512	2.14E-01	0.0146	Ture	0.1979%
rs6598541	15	99271135	A	G	184.9220666	-0.0114	0.0017	4.12E-12	0.6480	1.4970%	-0.0114	0.0017	4.12E-12	0.6480	Ture	1.4970%	-0.0079	0.0097	4.17E-01	0.6441	Ture	0.1283%	-0.0068	0.0122	5.78E-01	0.6439	Ture	0.0888%
rs72886778	2	169815106	T	A	158.4035596	0.0693	0.0124	1.73E-08	0.0140	1.2476%	0.0693	0.0124	1.73E-08	0.0140	Ture	1.2476%	-0.0386	0.0484	4.25E-01	0.9890	Ture	0.1256%	-0.0398	0.0602	5.08E-01	0.9890	Ture	0.1053%
rs73018724	2	169815248	A	G	269.5391893	0.0528	0.0056	1.35E-21	0.0450	2.1046%	0.0528	0.0056	1.35E-21	0.0450	Ture	2.1046%	-0.0295	0.0209	1.58E-01	0.0516	Ture	0.2223%	0.0113	0.0265	6.70E-01	0.0512	Ture	0.0679%
rs76031050	2	169816670	G	A	278.6047073	-0.0560	0.0055	1.49E-24	0.0290	2.2726%	-0.0560	0.0055	1.49E-24	0.0290	Ture	2.2726%	0.0122	0.0233	5.99E-01	0.0412	Ture	0.0825%	-0.0264	0.0293	3.67E-01	0.0412	Ture	0.1436%
rs78896123	2	169865452	C	T	126.089682	0.0611	0.0137	5.48E-05	0.0140	0.9957%	0.0611	0.0137	5.48E-05	0.0140	Ture	0.9957%	0.0039	0.0385	9.20E-01	0.0150	Ture	0.0160%	-0.0185	0.0486	7.03E-01	0.0150	Ture	0.0607%

Supplementary Table 4. Colocalization analysis of the drug effects of insulin (exposure) and OA (outcome).

Target gene	Outcome	Number of SNPs	$PP.H_0$	$PP.H_1$	$PP.H_2$	$PP.H_3$	$PP.H_4$
	OA	134	0	0.783	0	0.0967	0.12
LRP2	*KOA*	134	0	0.756	0	0.104	0.14
	HOA	134	0	0.738	0	0.00346	0.258
	OA	66	0.00705	0.799	0.000935	0.106	0.0876
IGF1R	*KOA*	66	0.00682	0.772	0.00103	0.116	0.104
	HOA	66	0.00669	0.758	0.0012	0.136	0.098

PP.H0, PP.H1, PP.H2, PP.H3 and PP.H4 represent the probability of no shared causal variant, a causal variant for exposure, a causal variant for outcome, distinct causal variants between two traits and a shared causal variant between two traits in the drug target gene.

Supplementary table 5. Detailed results of the valdation analyses of postive controls.

Outcome	Methods	OR	95% CI	P value	Heterogenity		Pleiotropy		MR-PRESSO global test	
					Q	P	Intercept	P	RSSobs	P
T2DM	IVW	0.6911	0.5499, 0.8687	1.54E-03	15.20476	0.4367711	-0.019642	0.0606693	16.95976	0.477
	MR Egger	1.0186	0.6584, 1.5760	9.35E-01						
	MR-PRESSO	0.6911	0.5499, 0.8687	6.38E-03						
	MR.RAPS	0.6950	0.5393, 0.8956	4.92E-03						
HbA1C	IVW	0.6531	0.6318, 0.6751	6.32E-140	21.42062	0.12392	0.0034453	0.0131051	23.81087	0.435
	MR Egger	0.6102	0.5778, 0.6443	5.31E-11						
	MR-PRESSO	0.6531	0.6318, 0.6751	1.09E-13						
	MR.RAPS	0.6498	0.6287, 0.6716	0.00E+00						

Supplementary table 6. Detailed results of the MVMR analyses.

Exposures and mediators	Outcome	IVW		MR-Egger		Heterogenity		Pleiotropy			Conditional F
		OR (95%CI)	P value	OR (95%CI)	P value	Q	P	Intercept	SD	P	
Insulin		1.5872 (1.1600, 2.1719)	0.004	1.2498 (0.8281, 1.8863)	0.289						2.7430
SBs		2.0483 (0.1357, 30.9261)	0.605	1.2930 (0.0817, 20.4634)	0.855						5.6377
ACL		0.8171 (0.3709, 1.8002)	0.617	0.9724 (0.4311, 2.1933)	0.947						5.8529
SMO	OA	16.1998 (3.6884, 71.1507)	0.000	12.0733 (2.6484, 55.0389)	0.001	8.551	0.999	0.009	0.005	0.080	3.0334
AGE		1.0544 (0.6224, 1.7865)	0.843	1.0450 (0.6168, 1.7705)	0.870						2.5850
BMI		0.3107 (0.1848, 0.5223)	0.000	0.6894 (0.2454, 1.9366)	0.480						16.3285
SEX		0.3926 (0.0130, 11.8400)	0.591	0.2099 (0.0065, 6.8073)	0.379						4.5779
Insulin		1.4579 (0.9948, 2.1366)	0.054	1.1560 (0.6986, 1.9131)	0.573						2.7430
SBs		6.3471 (0.2299, 175.2616)	0.275	4.0552 (0.1385, 118.7571)	0.416						5.6377
ACL		0.8504 (0.3236, 2.2351)	0.743	1.0070 (0.3721, 2.7256)	0.989						5.8529
SMO	KOA	17.6723 (2.9005, 107.6753)	0.002	13.3031 (2.0912, 84.6259)	0.006	6.3625	0.999	0.008	0.006	0.162	3.0334
AGE		1.2238 (0.6422, 2.3323)	0.538	1.2129 (0.6365, 2.3114)	0.558						2.5850
BMI		0.2590 (0.1372, 0.4887)	0.000	0.5638 (0.1596, 1.9922)	0.373						16.3285
SEX		0.3697 (0.0058, 23.6679)	0.639	0.2007 (0.0029, 14.0317)	0.459						4.5779
Insulin		1.6438 (1.0169, 2.6570)	0.043	1.5450 (0.8219, 2.9041)	0.177						2.7430
SBs		0.0662 (0.0010, 4.2298)	0.201	0.0588 (0.0009, 4.0377)	0.189						5.6377
ACL		1.3854 (0.4126, 4.6519)	0.598	1.4492 (0.4158, 5.0507)	0.560						5.8529
SMO	HOA	9.6794 (1.0003, 93.6608)	0.050	8.9710 (0.8776, 91.7034)	0.064	3.3503	0.999	0.002	0.007	0.766	3.0334
AGE		0.9085 (0.4051, 2.0371)	0.815	0.9057 (0.4039, 2.0310)	0.810						2.5850
BMI		0.7233 (0.3264, 1.6028)	0.425	0.8896 (0.1829, 4.3263)	0.885						16.3285
SEX		1.0942 (0.0059, 202.6418)	0.973	0.9287 (0.0045, 191.9433)	0.978						4.5779

MVMR: mutivariable mendelian randomization; OR:odds ration; CI: credential intervals; SBs: sedentary behaviors; ACL: alcohol intake; SMO: cigarette consumption; AGE: age at recruitment; BMI: body maSBs index; SEX: genetic sex.
