# Supplementary material for: Genetic proxies for therapy of insulin drug targets and risk of osteoarthritis: a drug-target Mendelian randomization analysis
Source: Inflammopharmacology. 2024 Aug 11;32(6):3717–28. doi: 10.1007/s10787-024-01542-8 (PMC11550247; doi:10.1007/s10787-024-01542-8)
Supplement: Supplementary file 2 — Supplementary file2 (PDF 1319 KB) [file 10787_2024_1542_MOESM2_ESM.pdf]

Supplementary Figure 1. Leave-one-out stability tests of the univariable mendelian randomization analysis of the effect of insulin and osteoarthiritis. Calculate the MR results of the remaining IVs after removing the IVs one by one. (A) The effect on overall OA; (B) The effect on KOA; (C) The effect on HOA.

Supplementary Figure 2. Scatter plots of the univariable mendelian randomization analysis of the effect of insulin and osteoarthiritis. The slope of each line corresponding to the estimated MR effect in different models, including the conventional IVW, BWMR, WMM, MR-Egger, MR-RAPS and MR-PRESSO methods. (A) The effect on overall OA; (B) The effect on KOA; (C) The effect on HOA.

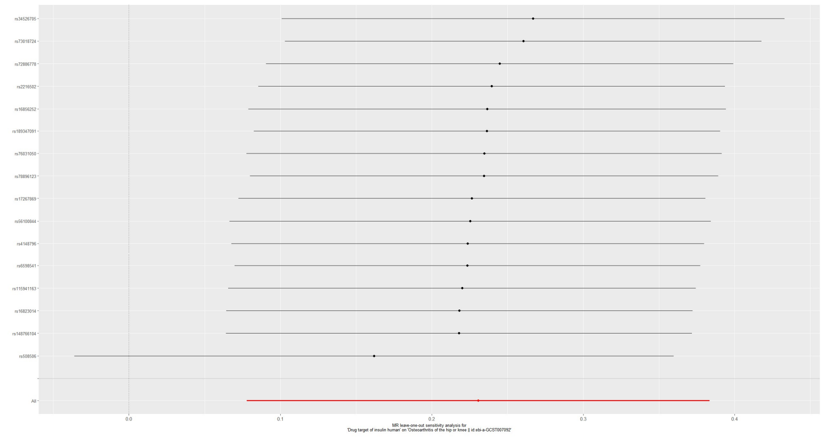

(A)

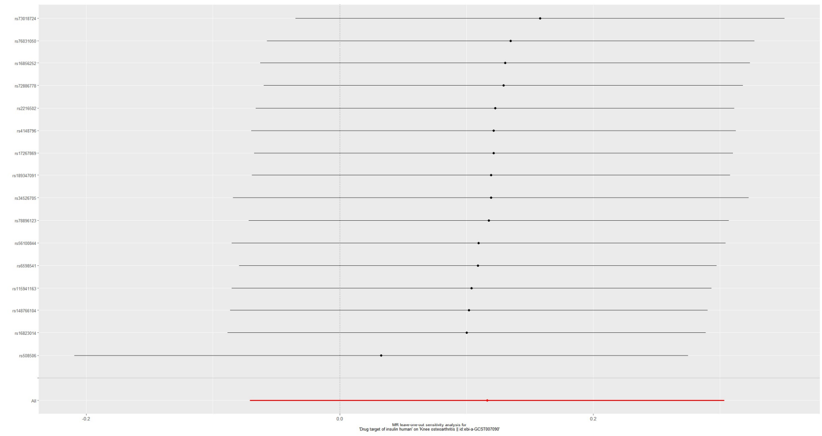

(B)

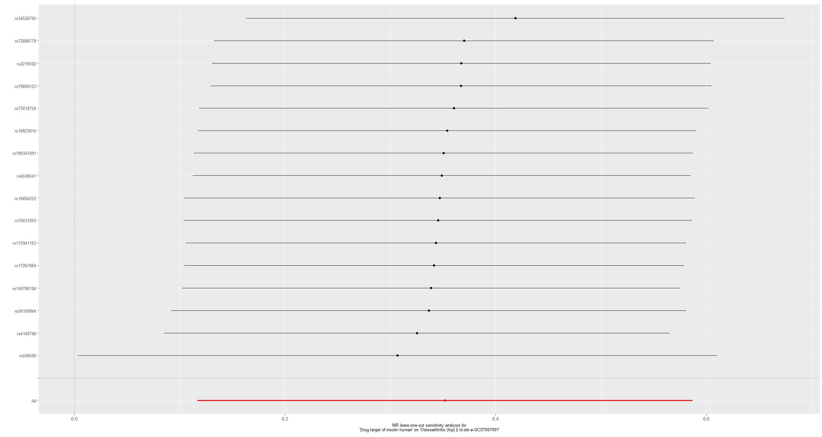

(C)

Supplementary Figure 1. Leave-one-out stability tests of the univariable mendelian randomization analysis of the effect of insulin and osteoarthiritis. Calculate the MR results of the remaining IVs after removing the IVs one by one. (A) The effect on overall OA; (B) The effect on KOA; (C) The effect on HOA.

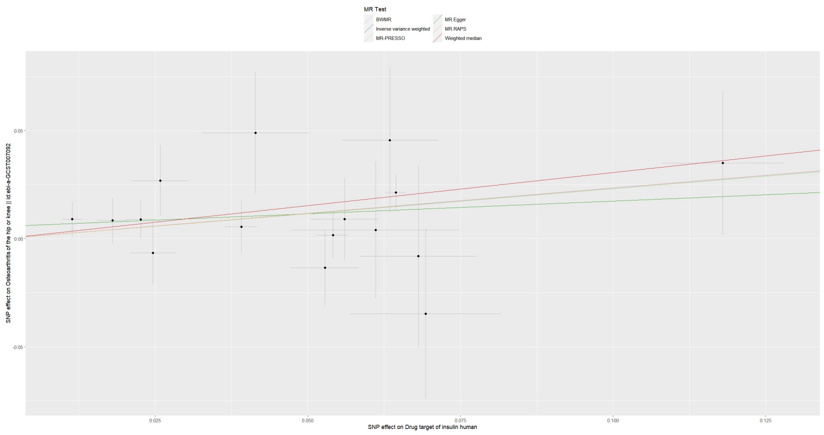

(A)

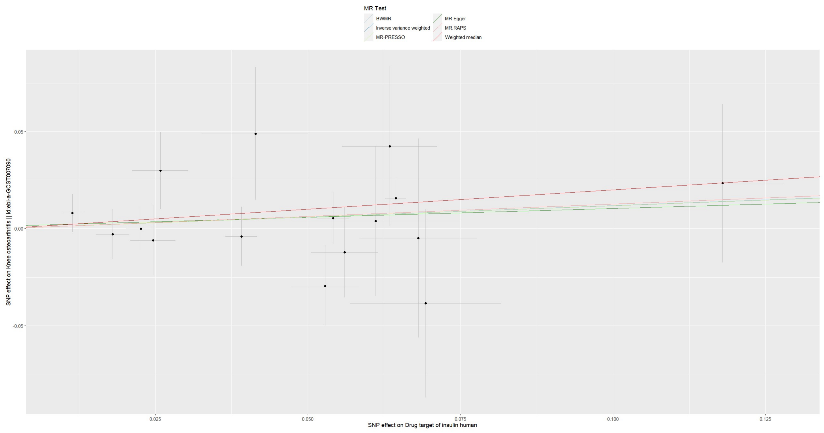

(B)

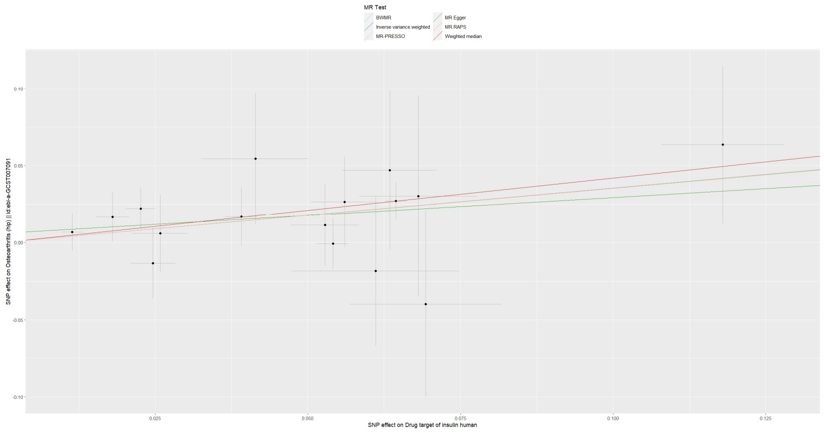

(C)

Supplementary Figure 2. Scatter plots of the univariable mendelian randomization analysis of the effect of insulin and osteoarthiritis. The slope of each line corresponding to the estimated MR effect in different models, including the conventional IVW, BWMR, WMM, MR-Egger, MR-RAPS and MR-PRESSO methods. (A) The effect on overall OA; (B) The effect on KOA; (C) The effect on HOA.
